# A transcriptomic analysis of skeletal muscle tissues reveals promising candidate genes and pathways accountable for different daily weight gain in Hanwoo cattle

**DOI:** 10.1038/s41598-023-51037-9

**Published:** 2024-01-03

**Authors:** Sunirmal Sheet, Sun Sik Jang, Jae Hwan Kim, Woncheoul Park, Dahye Kim

**Affiliations:** 1 Animal Genomics and Bioinformatics Division, Rural Development Administration, National Institute of Animal Science, Wanju, 55365 Republic of Korea; 2 Hanwoo Research Institute, National Institute of Animal Science, RDA, Pyeongchang, 25342 Republic of Korea

**Keywords:** Bioinformatics, Sequencing

## Abstract

Cattle traits like average daily weight gain (ADG) greatly impact profitability. Selecting based on ADG considering genetic variability can lead to economic and genetic advancements in cattle breeding. This study aimed to unravel genetic influences on ADG variation in Hanwoo cattle at the skeletal muscle transcriptomic level. RNA sequencing was conducted on longissimus dorsi (LD), semimembranosus (SB), and psoas major (PM) muscles of 14 steers assigned to same feed, grouped by low (≤ 0.71 kg) and high (≥ 0.77 kg) ADG. At P ≤ 0.05 and log2fold > 1.5, the distinct pattern of gene expression was identified with 184, 172, and 210 differentially expressed genes in LD, SB, and PM muscles, respectively. Tissue-specific responses to ADG variation were evident, with myogenesis and differentiation associated JAK-STAT signaling pathway and prolactin signaling pathways enriched in LD and SB muscles, while adipogenesis-related PPAR signaling pathways were enriched in PM muscle. Key hub genes (*AXIN2*, *CDKN1A*, *MYC*, *PTGS2*, *FZD5*, *SPP1*) were upregulated and functionally significant in muscle growth and differentiation. Notably, *DPP6*, *CDKN1A*, and *FZD5* emerged as possible candidate genes linked to ADG variation. These findings enhance our understanding of genetic factors behind ADG variation in Hanwoo cattle, illuminating skeletal muscle mechanisms influencing ADG.

## Introduction

An indigenous Korean native cattle breed called Hanwoo (*Bos taurus coreanae*) has been heavily bred for producing meat due to their quick growth and high-quality meat over the past 30 years. In Hanwoo breeds, the emphasis is placed on maximizing the efficiency of weight gain while minimizing fat deposition^1^. Here, the daily body weight gain (ADG) serves as an important indicator of the eventual carcass weight, with less emphasis on fat content. In commercial beef production, ADG of Hanwoo cattle is one of the significant components that affects the perception of meat price. This factor should be taken into consideration in genetic improvement programs as part of the evaluation of the economic return on investment in beef cattle production.

Moreover, genetic and environmental factors play a significant role in determining the different daily weight gain, which contributes to the overall carcass weight variation^1,2^. Daily weight gain is a complex carcass trait and the heritability of average daily weight gain in Korean native Hanwoo cattle is reported to be moderate to high^3,4^. Studies have estimated the heritability of this trait to be around 0.30 to 0.60^3,4^. This suggests that a considerable portion of the variation in average daily weight gain can be attributed to genetic factors, indicating the potential for selective breeding to improve this trait in the Hanwoo cattle population. Therefore, we focused on precise genetic mechanism underlying behind ADG variations in Hanwoo cattle.

A comprehensive understanding of the genetic mechanisms underlying key growth and livestock traits paves the way for identifying new genes and genetic pathways which would be beneficial for genomic selection in livestock species. Over the past decade, it has been a prevailing approach to carry out transcriptomic investigations on small-scale experimental cohorts, with the objective of elucidating the genetic components involved in the intricate biological processes associated with complex traits and diseases^5–8^. RNA-Sequencing (RNA Seq) has emerged as a preferred method over previous techniques like microarrays due to its ability to capture the entire transcriptome, including novel transcripts, alternative splicing events, and non-coding RNAs. Moreover, it has been suggested that the patterns of gene expression show higher levels of concordance across various breeds and populations, when compared to other analysis for example genome-wide association studies (GWAS)^5,6^.

Most earlier studies employed GWAS-based methods to analyze the genetic basis of carcass traits, revealing several genomic regions containing QTLs associated with carcass weight in diverse cattle breed^7,9,10^. However, the investigation of carcass traits in Hanwoo cattle through transcriptomic studies, employing RNA sequencing for functional evaluation of pivotal candidate genes, is still in a dynamic phase and requires more comprehensive analyses.

In the present study, we conducted a transcriptome analysis on three specific skeletal muscles—longissimus dorsi muscle (LD), semimembranosus (SB), and psoas major (PM). These samples were collected from 14 steers, divided into two groups based on their ADG, in order to gain a comprehensive understanding of the genetic mechanisms underlying this complex trait and explore potential candidate genes or molecules involved. We performed functional enrichment and pathway analyses on the differentially expressed genes (DEGs) to identify significantly enriched gene ontology (GO) terms and Kyoto Encyclopedia of Genes and Genomes (KEGG) pathways associated with ADG. Additionally, we constructed a protein–protein interaction (PPI) network to identify hub genes based on their connectivity within the network.

## Results

**Figure 1:**
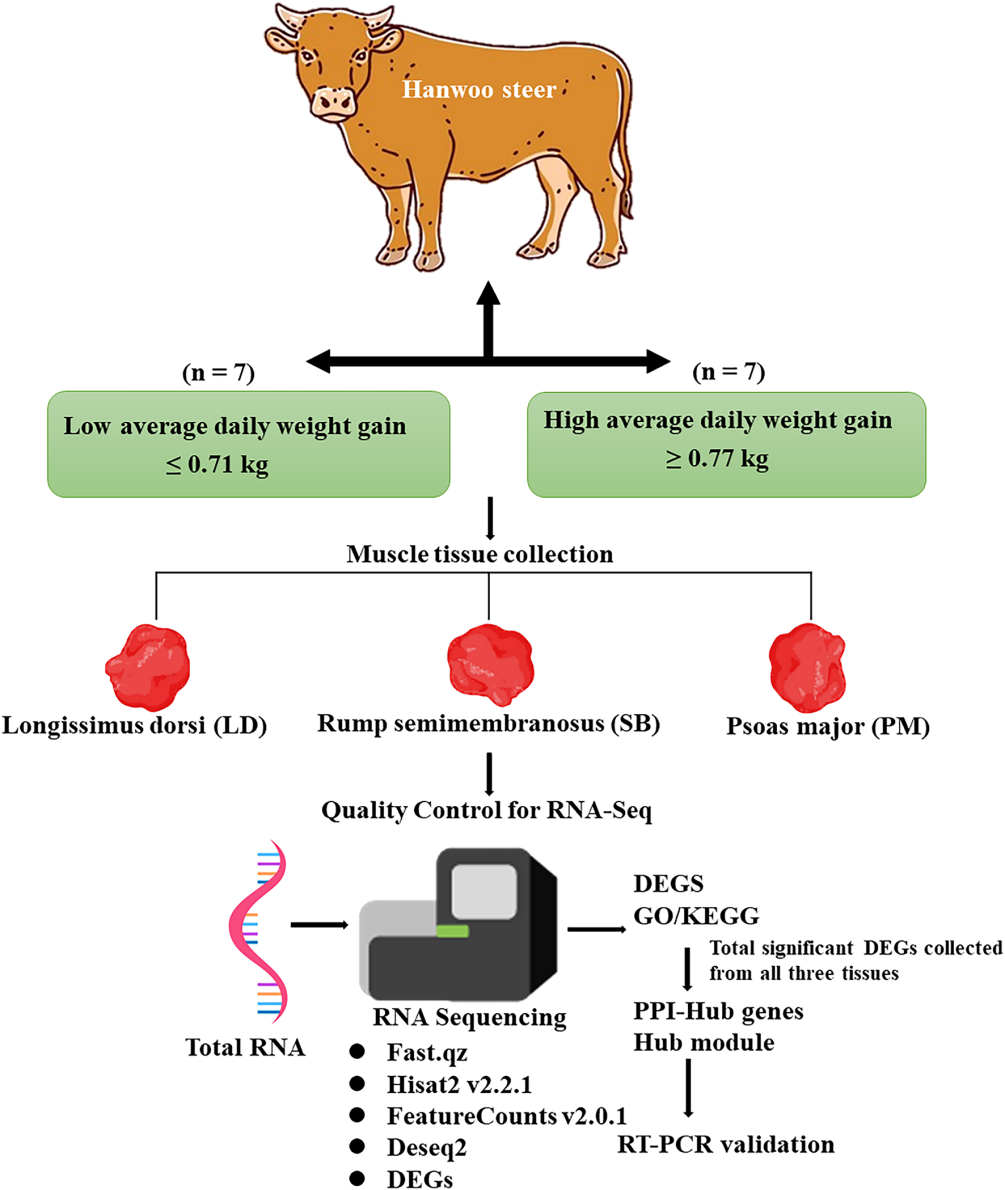
The diagram illustrates the experimental design. *DEGs* differentially expressed genes, *GO* Gene Ontology, *KEGG* Kyoto Encyclopedia of Genes and Genomes, *PPI* protein–protein interaction.

A cohort of 14 Hanwoo steers that were divided into two ADG groups—high ADG (≥ 0.77 kg, 7 steers) and low ADG (≤ 0.71 kg, 7 steers) were the subject of a transcriptome investigation. A full summary of our study is summarized in Fig. 1. The significant difference (P < 0.001) of ADG between two group was shown in Supplementary Fig. 1. The details regarding the animals, including their phenotype, age, and sex, can be found in Table 1 of the study.

**Table 1:** Summary of phenotypic data of animals used in this study (n = 14).

Group	No of animal (n = 7)	Sex	Born weight (kg)	Wight at Slaughter (kg)	Weight gain (kg)	Age (days)	ADG*** (kg/d)	Tissue collected
Low-ADG ≤ 0.71 kg	212,004	M	28	228	200	356	0.56	Longissimus dorsi muscle (LD), Semimembranosus (SB), Psoas major (PM)
	212,003	M	25	250	225	357	0.63	
	214,031	M	27	317	290	431	0.67	
	204,006	M	20	243	223	328	0.68	
	212,008	M	19	289	270	388	0.70	
	202,039	M	25	354	329	461	0.71	
	202,037	M	26	314	288	403	0.71	
High-ADG ≥ 0.77 kg	192,033	M	34	273	239	311	0.77	
	212,002	M	34	424	390	472	0.83	
	192,023	M	32	428	396	447	0.89	
	212,012	M	23	467	444	479	0.93	
	192,020	M	27	450	423	450	0.94	
	212,021	M	26	481	455	455	1.00	
	212,019	M	35	375	340	347	0.98	

*ADG* Average daily weight gain.

***p < 0.001 indicate significant difference of ADG between Low-ADG and High-ADG group.

### Transcriptome analysis

**Figure 2:**
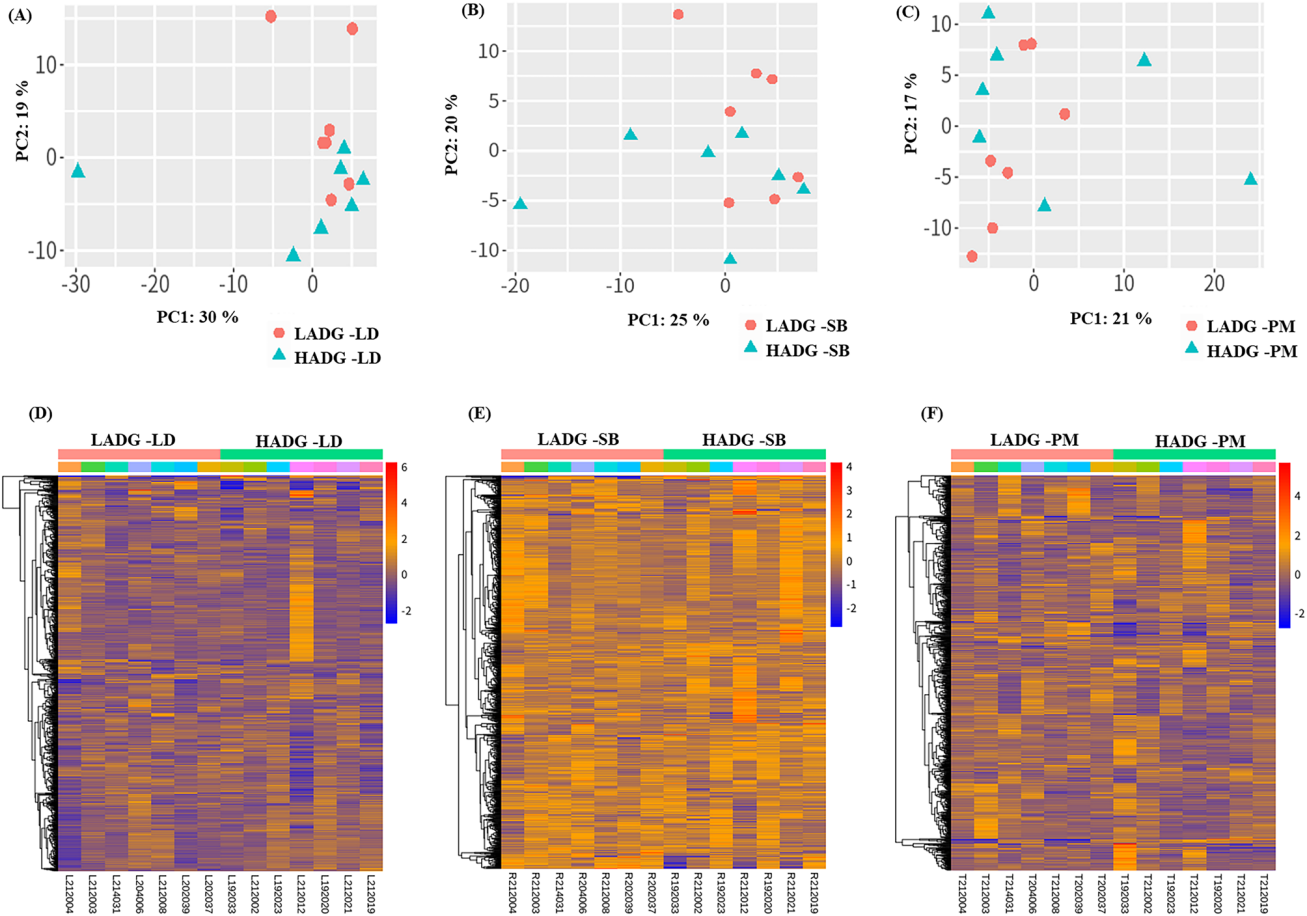
(**A**) Data preprocessing and differential expression gene (DEG) analysis on the LD, SB, and PM muscle tissue samples from low and high average daily weight gain Hanwoo cattle. Principal component analysis indicating the overall profiles of three muscles datasets (**A**) LD, (**B**) SB, and (**C**) PM. Heatmap of the top 500 DEGs of (**D**) LD, (**E**) SB, and (**F**) PM muscles at P value < 0.05 and log2FC > 1.5 and column represents the gene expression pattern of each sample. Up- and down-regulated transcripts are shown in red and blue color reFigurespectively. *LADG* low average daily weight gain, *HADG* high average daily weight gain, *LD* longissimus dorsi, *SB* semimembranosus, *PM* psoas major.

In this study, RNA was extracted from specific three skeletal muscle tissue samples, including LD, SB, and PM muscle tissues. The extracted RNA was then used to construct libraries for deep sequencing. After sequencing, the obtained sequences were utilized for further analysis. Finally, we determined the total DEGs genes in each tissue, as presented in Supplementary File S1. In order to detect any underlying patterns in the samples, a principle component analysis (PCA) was carried out on DEGs without applying batch correction. The analysis effectively separated the samples of high ADG group from those the low ADG group samples (Fig. 2A–C). The results demonstrated that the first principal component (PC1) explained 30% of the variability, while PC2 explained 19% of the variability within the DEGs of LD samples, as shown in Fig. 2. For SB tissue, PC1 explained 21% of the variability, whereas PC2 explained 17% of the variability. For PM tissue, it was 25% of the variability of PC1, and 20% variability for PC2.

### DEGs identification

After conducting statistical analysis using DESeq2 on the gene expression matrix, we identified a total of 15,473 gene symbols for LD, 15,790 genes symbols for SB and 15,775 gene symbols for PM groups. Among them, 184 genes for LD, 172 for SB and 210 genes for PM were found to be differentially expressed when comparing low ADG and high ADG samples. These DEGs consisted of 66 up-regulated genes and 11 down-regulated genes for LD, 107 up-regulated genes and 65 down-regulated gene for SB and 65 up-regulated genes and 145 down regulated genes for PM group (Supplementary File S1). In SB samples, we found the number of differentially upregulated gene is higher compared to other two groups. The criteria for determining DEGs were a significance threshold P-value of ≤ 0.05 and a minimum log2 fold change of 1.5. A list of top 10 upregulated and downregulated gene is provided in Table 2. For LD, we found *DPP6*, *PIPOX*, *ENSBTAG00000019227*, *FRMPD3*, *ENSBTAG00000046937*, *U6*, *BHMT2*, *ERICH6*, *C15H11orf94*, and *KCNC3* were the top upregulated gene and *ZDHHC22*, *ENSBTAG00000001308*, *DRC1*, *CDKL*, *ENSBTAG00000006239*, *MFRP*, *PLPPR3*, *ENSBTAG00000047538*, *C3H1orf185*, and *U3* were the top downregulated gene. The genes *IL22RA1*, *CLDN7*, *EHF*, *SLC34A2*, *PITX1*, *U2*, *RNF180*, *LRATD1*, *TP53TG5*, and *SLC45A4* identified as top upregulated characterized gene and *KCNG2*, *STMN2*, *OTOGL*, *U3*, *MYL6B*, *FGF6*, *U3*, *ACSBG1*, *TPC3*, and *KRT19* downregulated for SB tissue. In PM tissue, *U6*, *SRARP*, *CSTB*, *TUBB3*, *PADI4*, *DSG4*, *ISG20*, *SULT1C3*, *VXN*, and *SPP1* were top upregulated genes and *MPTX1*, *MGAM*, *PIGR*, *WNT16*, *GNLY*, *AZGP1*, *CKMT1A*, *MLANA*, *FOXQ1*, and *SUSD5* were top downregulated genes.

**Table 2:** The top10 upregulated DEGs and downregulated DEGs detected between low-ADG and high-ADG groups with log2fold change ≥ 1.5 and P < 0.05.

Upregulated DEGs			Downregulated DEGs		
Gene	FC	P value	Gene	FC	P value
LD tissue					
DPP6	4.39	0.017	ZDHHC22	− 3.65	0.022
PIPOX	4.24	0.002	DRC1	− 3.41	0.035
FRMPD3	3.99	0.001	CDKL4	− 3.37	0.011
U6	3.32	0.004	MFRP	− 3.23	0.033
BHMT2	3.08	0.020	PLPPR3	− 3.10	0.006
ERICH6	2.91	0.031	C3H1orf185	− 3.02	0.029
C15H11orf94	2.79	0.004	U3	− 2.99	0.001
KCNC3	2.75	0.037	NUDT11	− 2.95	0.012
ADGRE3	2.71	0.047	GLRB	− 2.89	0.003
TDRKH	2.69	0.025	SNORA73	− 2.86	0.001
SB tissue					
IL22RA1	4.15	0.031	KCNG2	− 2.466	0.001
CLDN7	3.89	0.021	STMN2	− 2.376	0.010
EHF	3.59	0.001	OTOGL	− 2.159	0.001
SLC34A2	3.46	0.043	U3	− 2.009	0.030
PITX1	2.89	0.001	MYL6B	− 1.931	0.009
U2	2.81	0.008	FGF6	− 1.845	0.029
RNF180	2.61	0.030	U3	− 1.827	0.033
LRATD1	2.14	0.018	ACSBG1	− 1.813	0.009
TP53TG5	2.12	0.002	TPC3	− 1.785	0.024
SLC45A4	2.06	0.002	KRT19	− 1.715	0.048
PM tissue					
U6	3.99	0.005	MPTX1	− 5.53	0.044
SRARP	3.91	0.046	MGAM	− 4.08	0.002
CSTB	3.43	0.010	PIGR	− 3.99	0.001
TUBB3	3.40	0.032	WNT16	− 3.31	0.006
PADI4	3.40	0.032	GNLY	− 3.21	0.014
DSG4	3.17	0.011	AZGP1	− 3.20	0.002
ISG20	3.09	0.009	CKMT1A	− 3.17	0.032
SULT1C3	2.94	0.007	MLANA	− 3.00	0.011
VXN	2.89	0.008	FOXQ1	− 2.95	0.033
SPP1	2.80	0.010	SUSD5	− 2.94	0.012

*FC* fold change, *LD* longissimus dorsi muscle, *SB* semimembranosus, *PM* psoas major.

**Figure 3:**
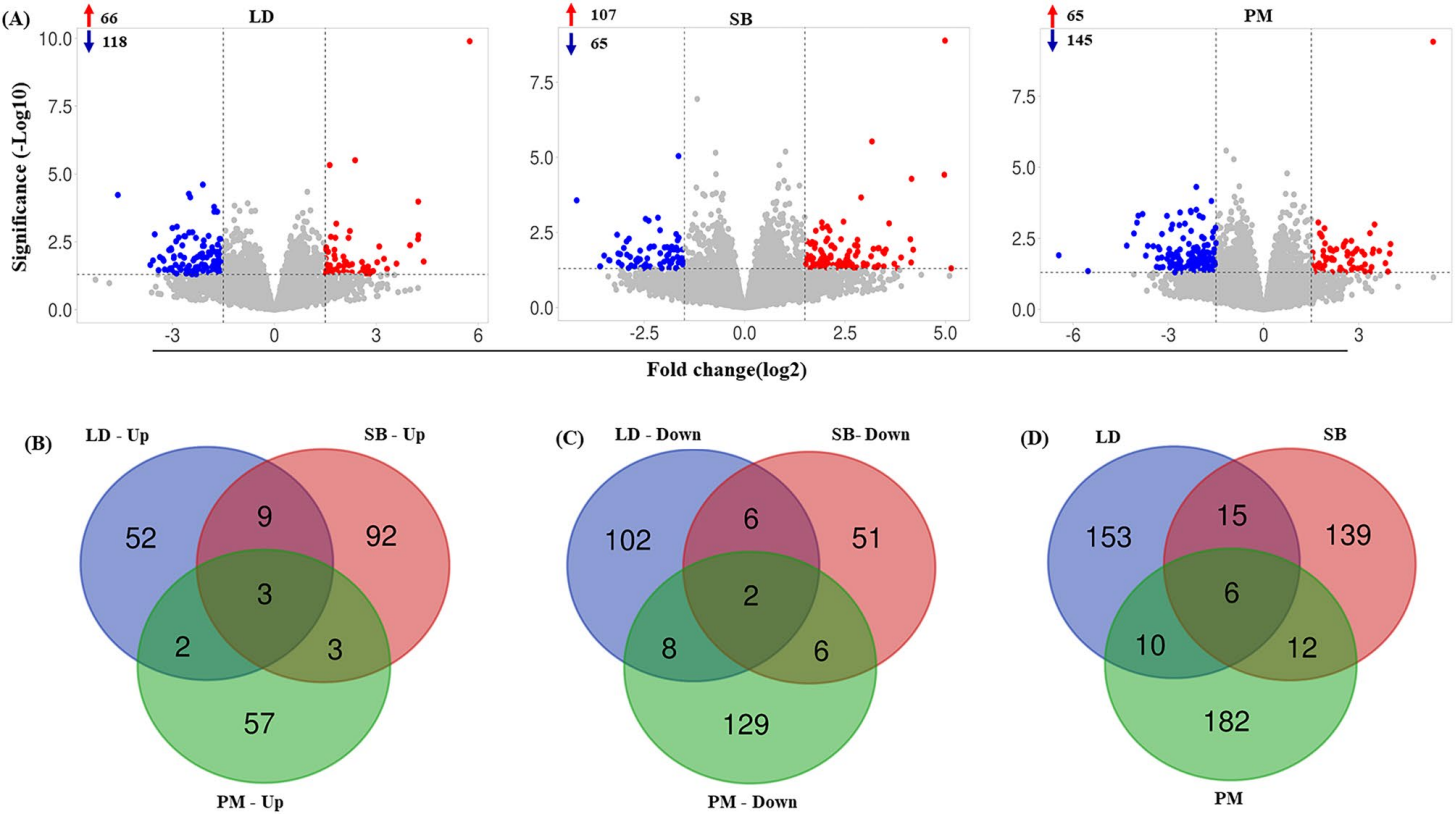
(**A**) Volcano plots visualizing significant DEGs with P ≤ 0.05 and log2FC ≥ 1.5 between low and high average daily weight gain (ADG) Hanwoo cattle muscle tissue samples. Red points symbolize up-regulation, while blue points designate down-regulation; gray points signify non-significant expression. The number of up- and down- regulated genes presented as red arrow and blue arrow symbol respectively. A Venn plot to identify common (**B**) up-regulated, (**C**) down-regulated, (**D**) total genes among the muscle tissue samples. *LD* longissimus dorsi, *SB* semimembranosus, *PM* psoas major, *FC* fold change.

The expression pattern of DEGs across samples was analyzed using hierarchical clustering. The results revealed that the gene expression of the low ADG group was significantly different from that of the samples of high ADG group in all the tissues. Heatmaps were generated to visualize the expression patterns of the top 500 DEGs in each tissue of two different ADG group (Fig. 2D–F). To further investigate the differential gene expression, we generated a volcano plot (Fig. 3A). Venn diagrams were used to illustrate the overlap of upregulated and downregulated DEGs between the evaluated tissues (Fig. 3B–D). The Venn diagram shows only six genes were found to be overlapped. The correlation between the expression levels of tissues were shown in Supplementary Fig. 2.

### KEGG pathway and network analysis

**Figure 4:**
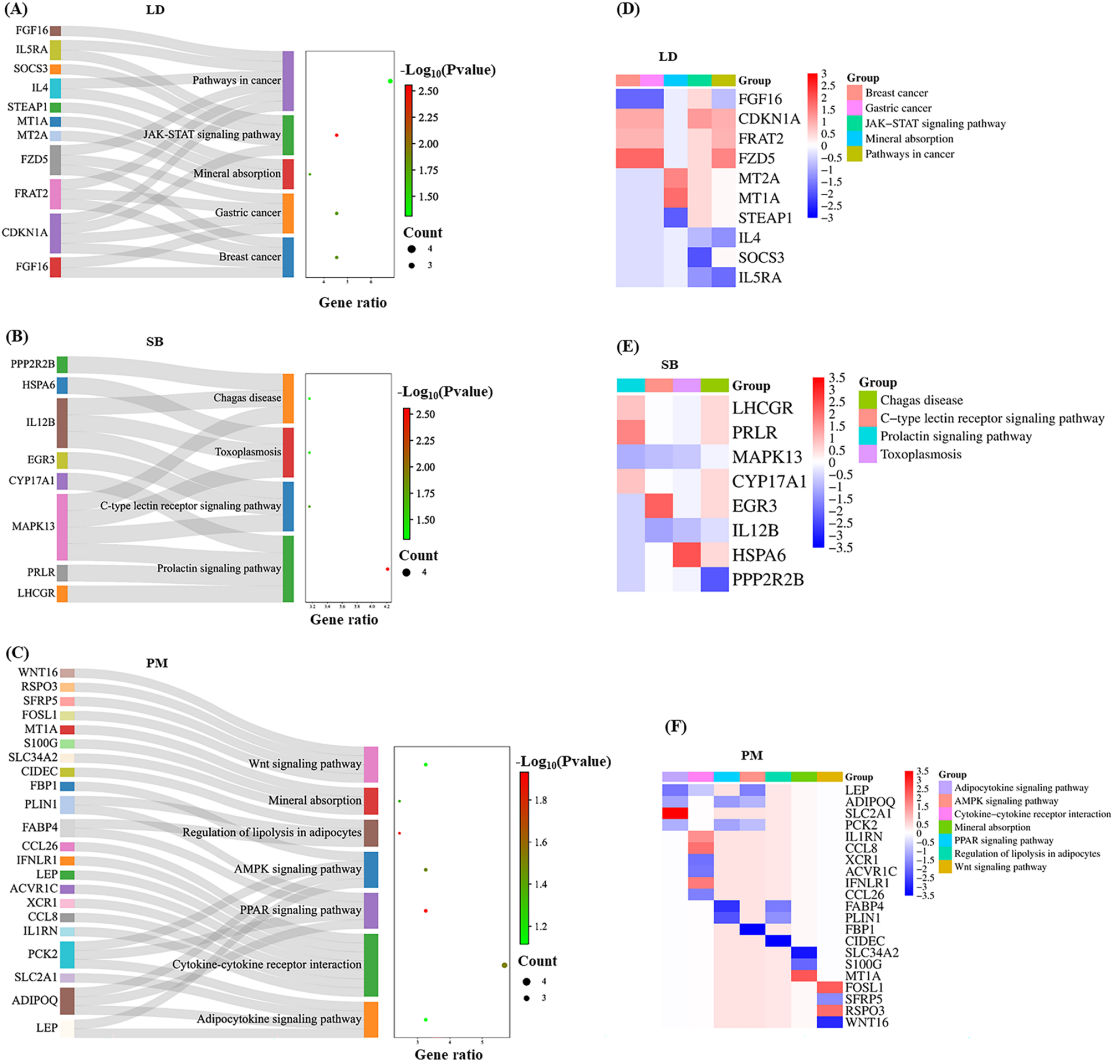
Function enrichment analysis of differently expressed genes. Sankey plot representing the each enriched KEGG pathway (P ≤ 0.05) for (**A**) LD, (**B**) SB, (**C**) PM tissues. Heatmap represents the expression of each gene associated with respectively enriched pathway in (**D**) LD, (**E**) SB, (**F**) PM muscle tissues. *LD* longissimus dorsi, *SB* semimembranosus, *PM* psoas major, *KEGG* Kyoto Encyclopedia of Genes and Genomes.

In this study, the KEGG pathway analysis successfully annotated the DEGs to a total of 16, 14, and 10 pathways for LD, SB, PM tissues (Fig. 4). KEGG pathway analysis of predicted DEGs targets featured enrichment for bta04630:JAK-STAT signaling pathway, bta05224:breast cancer, bta05226:gastric cancer, bta04978:mineral absorption, and bta05200:pathways in cancer in LD tissue for the SB tissue, we found the bta04917:prolactin signaling pathway, bta04625:c-type lectin receptor signaling pathway, bta05145:toxoplasmosis, and bta05142:chagas disease pathways to be significantly enriched. KEGG pathway analysis of predicted DEGs of PM tissue targets featured enrichment for bta04920:adipocytokine signaling pathway, bta03320:PPAR signaling pathway, bta04923:regulation of lipolysis in adipocytes, bta04060:cytokine-cytokine receptor interaction, bta04152:AMPK signaling pathway, bta04978:mineral absorption, bta04310:WNT signaling pathway. Figure 4 showcased that genes such as *IL4*, *FZD5*, *FRAT2*, *CDKN1A*, *FGF16* in LD (Fig. 4A), *IL12B* and *MAPK13* in SB (Fig. 4B), and *PLIN1*, *FABP4*, *PCK2*, *ADIPOQ* in PM (Fig. 4C) tissue were the one which are involved in multiple pathways regulation. The expression of genes involved in pathways for each tissue is shown in heatmap (Fig. 4D–F). The KEGG graph of top pathways found in each tissue were given in Supplementary Fig. 3. This analysis helps to identify the specific pathways or signaling networks that are potentially dysregulated in relation to the observed gene expression changes.

### GO enrichment and network analysis

**Figure 5:**
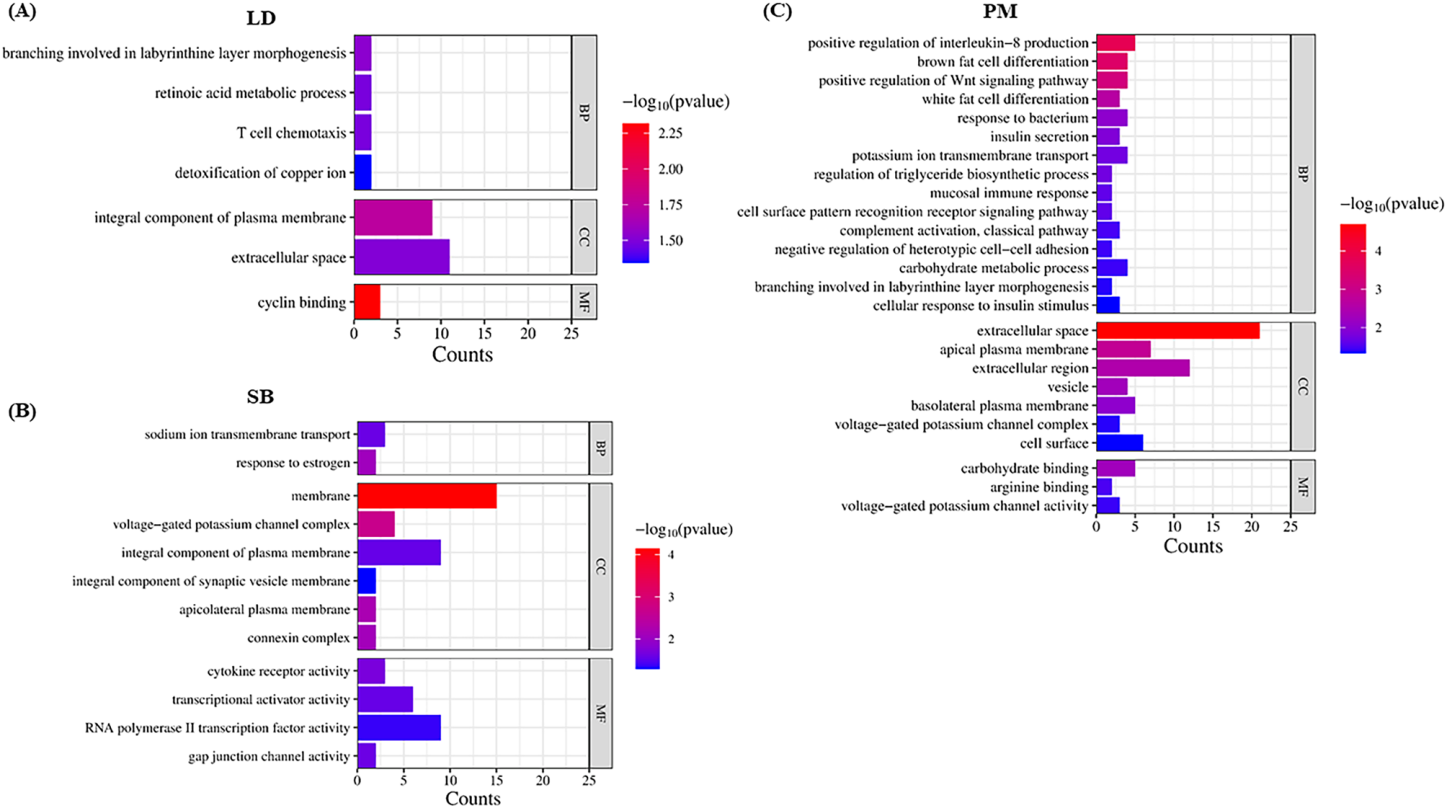
Function enrichment analysis of differently expressed genes. The significant (P ≤ 0.05) enriched gene ontology for (**A**) LD, (**B**) SB, (**C**) PM muscle tissues. *LD* longissimus dorsi, *SB* semimembranosus, *PM* psoas major.

**Figure 6:**
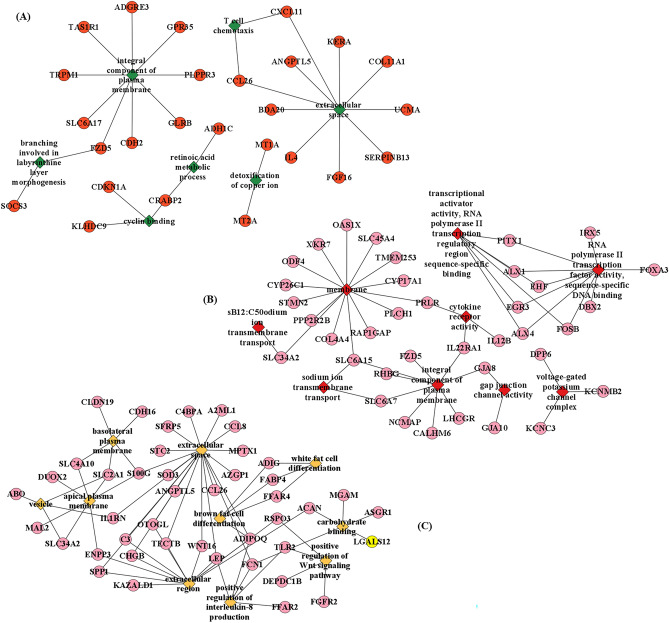
The network analysis of gene ontology of (**A**) LD, (**B**) SB, (**C**) PM muscle tissue samples showing genes involved in different gene ontology terms. *LD* longissimus dorsi, *SB* semimembranosus, *PM* psoas major.

To evaluate the functional relationships and biological changes associated with the identified DEGs, gene enrichment analysis using predetermined gene sets and gene ranks was conducted. Specifically, GO and KEGG pathway enrichment analyses were performed on the set of 186 (LD), 175 (SB) and 211 (PM) up-regulated and downregulated DEGs. The GO enrichment analysis revealed a total of 7 significant GO terms, with four terms significantly enriched in the biological process category, two terms significantly enriched in the cellular component category, and one terms significantly enriched in the molecular function category for LD tissue (Fig. 5A). For SB tissue, a total of 12 significant GO terms was identified in GO enrichment analysis. Among these, two terms showed significant enrichment in the biological process category, six terms exhibited significant enrichment in the cellular component category, and four terms displayed significant enrichment in the molecular function category (Fig. 5B). In PM tissue, a total of 25 significant GO terms found where 15 terms significantly enriched in the biological process category, seven terms significantly enriched in the cellular component category, and three terms significantly enriched in the molecular function category for PM tissue (Fig. 5C). These enriched GO terms provide insights into the functional roles and processes that the DEGs may be involved in. Further, all the significant GO terms were shown in Fig. 2. Here, we stated the top significant GO terms that were GO:0030332 ~ cyclin binding, GO:0016020 ~ membrane, GO:0005615 ~ extracellular space found to be associated with ADG variation in LD, SB, and PM tissue, respectively. In addition, we have constructed a network of the ten top mostly enriched GO terms for each tissue (Fig. 6).

### Hub genes, co-expression module and pathway detection by PPI network and module analysis of DEG

The PPI network was constructed using the STRING database, resulting in a network consisting of 363 nodes and 902 edges. Supplementary Fig. 4 provide visual representations of the network structure. The hub genes were selected for further investigation using PPI analysis. In order to identify hub genes for PPI analysis, all four methods within the CytoHubba plugin were utilized. The top 20 genes resulting from each method were compiled and presented in Table 3. Among these lists, a total of 12 genes that appeared in all four methods were considered as hub genes (*JUN*, *CDKN1A*, *CDH1*, *TP53*, *FZD5*, *PPARG*, *LEP*, *IL4*, *PTGS2*, *AXIN2*, *CDH2*, and *MYC*). These findings highlight the centrality of these hub genes and suggest their involvement in critical biological processes or pathways associated with growth performance in Hanwoo.

**Table 3:** The top 20 hub genes identified from protein–protein interaction network using the cytohubba plugin in cytoscape.

Ranking methods in the Cytohubba plugin							
Degree		EPC		MCC		MNC	
Gene	Score	Gene	Score	Gene	Score	Gene	Score
PPARG	36	SPP1	164.079	FOSL1	5041	CDKN1A	33
AXIN2	34	LEP	164.079	SOCS3	5041	IL4	31
CDKN1A	34	PPARG	164.079	TLR2	5041	MYC	31
IL4	31	IL4	164.079	PPARG	5040	JUN	29
MYC	29	CDKN1A	164.079	AXIN2	5040	TP53	28
FZD5	28	JUN	164.079	CDKN1A	5040	PPARG	27
PTGS2	28	CSF3	164.079	APOE	5040	AXIN2	26
CDH1	28	FZD5	164.079	CDH2	5040	CREBBP	25
TLR2	27	TP53	164.079	FZD5	4523	LEP	24
SPP1	22	MYC	164.079	PTGS2	4199	FZD5	21
JUN	21	CDH1	164.079	MYD88	3881	PTGS2	19
LEP	20	PTGS2	163.91	CDK2	3204	CSF3	19
CREBBP	20	AXIN2	163.795	IL4	3193	MAPK13	16
CSF3	18	MMP24	163.764	MYC	2786	SPP1	16
MAPK13	16	MAPK13	163.761	JUN	2483	CDH1	15
CDH2	16	CDH2	163.748	TP53	2477	TLR2	15
TP53	16	SOCS3	163.612	CREBBP	1724	CDH2	15
PPARGC1A	16	LIF	163.578	CDH1	1616	SOCS3	15
ACAN	15	FABP4	163.567	LEP	1543	EGR1	14
ADIPOQ	15	FGFR2	163.432	EGR1	1502	MMP24	14

*Degree* node connected degree, *EPC* edge percolated component, *MCC* maximal clique centrality, *MNC* maximum neighborhood component.

**Figure 7:**
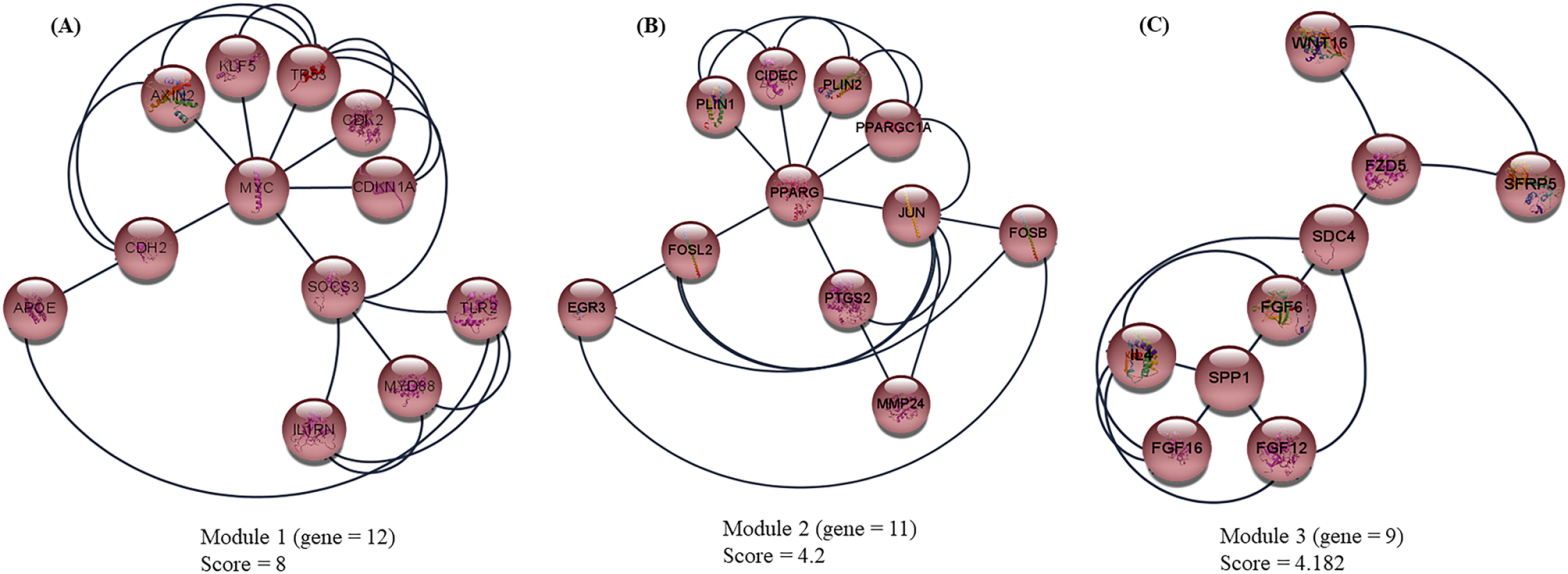
Module analysis from protein–protein interaction (PPI) network. Top three significant modules including (**A**) module 1, (**B**) module 2, and (**C**) module 3 were obtained from PPI network of differentially expressed genes with MCODE.

After conducting module analysis using the MCODE plugin in Cytoscape, a total of nineteen clusters were obtained. Based on their MCODE scores, the top three modules were selected as hub modules (Fig. 7). Among these modules, module 1 had the highest MCODE score (8) and consisted of 12 nodes and 23 edges. Module 2 was comprising 11 nodes and 24 edges with MCODE score 4.2. Next, the Module 3 had MCODE score of 4.182 and contained 9 nodes and 14 edges. Importantly, all genes within module 1 showed up-regulation, indicating their potential functional significance.

To gain further insights into the biological functions of the genes within each module, we performed a biological functional enrichment analysis using the DAVID database (Table 4). The genes in Module 1 showed significant enrichment in a total of 35 significant pathways. Here, we have highlighted here some of the top significant pathways such as PI3K-Akt signaling pathway, WNT signaling pathway, JAK-STAT signaling pathway, lipid and atherosclerosis, and MAPK signaling pathway. Module 2 including 11 genes was found to be associated with different biological pathways. Among them, tissue growth associated pathways were osteoclast differentiation, regulation of lipolysis in adipocytes, PPAR signaling pathway, IL-17 signaling pathway, and C-type lectin receptor signaling pathway. Module 3 consisting of 9 genes was noticed to be linked to important pathways including PI3K-Akt signaling pathway and Wnt signaling pathway. In addition, the expression level of genes found in all three modules are presented in Table 5.

**Table 4:** The biological pathway enrichment analysis of genes from the three modules of protein–protein interaction network.

Modules	Enriched pathway	P-value	Nodes/Genes
Module 1 (Gene = 12)			
APOE, AXIN2, CDH2, CDK2, CDKN1A, IL1RN, KLF5, MYC, MYD88, SOCS3, TP53, TLR2	Hepatitis B	9.91E − 07	CDKN1A, MYC, CDK2, TP53, MYD88, TLR2
	Epstein-Barr virus infection	4.02E − 06	CDKN1A, MYC, CDK2, TP53, MYD88, TLR2
	PI3K-Akt signaling pathway	2.11929E − 05	CDKN1A, MYC, CDK2, TP53, TLR2
	Pathways in cancer	3.36068E − 05	CDKN1A, MYC, CDK2, AXIN2, TP53
	p53 signaling pathway	3.85284E − 05	CDKN1A, CDK2, TP53
	Wnt signaling pathway	0.000132004	MYC, AXIN2, TP53
	Transcriptional misregulation in cancer	0.000155306	CDKN1A, MYC, TP53
	JAK-STAT signaling pathway	0.000615968	SOCS3, CDKN1A, MYC
	Lipid and atherosclerosis	7.17E − 04	TP53, MYD88, TLR2
	MAPK signaling pathway	7.44E − 04	MYC, TP53, MYD88
Module 2 (Gene = 11)			
CIDEC, EGR3, FOSB, FOSL2, JUN, MMP24, PLIN1, PLIN2, PPARG, PPARGC1A, PTGS2	Osteoclast differentiation	3.55E − 04	JUN, FOSB, PPARG, FOSL2
	Regulation of lipolysis in adipocytes	0.001721137	PLIN1, PTGS2, CIDEC
	PPAR signaling pathway	0.003376351	PLIN2, PPARG, PLIN1
	IL-17 signaling pathway	0.004309484	JUN, FOSB, PTGS2
	C-type lectin receptor signaling pathway	0.005349286	JUN, EGR3, PTGS2
	Thermogenesis	0.026003092	PPARG, PLIN1, PPARGC1A
Module 2 (Gene = 9)			
FGF12, FGF16, FGF6, FZD5, IL4, SDC4, SFRP5, SPP1, WNT16	Breast cancer	1.37E − 04	FGF6, FGF16, FZD5, WNT16
	Gastric cancer	1.45E − 04	FGF6, FGF16, FZD5, WNT16
	Pathways in cancer	3.48E − 04	IL4, FGF6, FGF16, FZD5, WNT16
	PI3K-Akt signaling pathway	0.002075303	IL4, FGF6, FGF16, SPP1
	Wnt signaling pathway	0.006710108	FZD5, SFRP5, WNT16
	Proteoglycans in cancer	0.009339395	FZD5, SDC4, WNT16
	Human papillomavirus infection	0.026411276	FZD5, SPP1, WNT16

**Table 5:** Expression level of genes found in three modules in all the tissues.

Module	Gene	LD	SB	PM
Module 1	Log2FC			
	APOE	− 0.243491288	− 0.027140434	− 1.021402425
	**AXIN2**^**#**^	0.599064633	**1.269194423**	0.321090503
	CDH2	− 1.667784418	0.826919592	0.094833306
	CDK2	− 0.322243936	− 0.130990721	0.157372361
	**CDKN1A**^**#**^	**2.220807551**	**1.240616518**	**1.018737006**
	IL1RN	− 0.340157462	− 0.415273494	**1.751094218**
	**KLF5**	0.546259975	**1.100195095**	0.64574664
	**MYC**^**#**^	0.823322049	0.908689108	**1.49625797**
	MYD88	0.219930853	0.267832958	− 0.065811156
	SOCS3	− 1.766991991	0.472539365	0.909865139
	TP53	0.447076635	0.558059046	0.102911973
	TLR2	0.783592371	0.127463344	**1.533538354**
Module 2	CIDEC	− 0.768653672	0.927102646	− 1.820372372
	**EGR3**	0.008927441	**1.638251716**	0.659959821
	**FOSB**	− 1.391513882	**1.894382261**	0.546207447
	**FOSL2**	0.564164172	**1.017327673**	0.596711166
	JUN	− 0.048373012	0.607334123	0.135294738
	MMP24	− 0.105063312	− 0.220427562	− 1.300511674
	PLIN1	− 0.3718207	0.845443235	− 1.728098676
	**PLIN2**	0.482058292	0.258884573	**1.088125607**
	PPARG	− 0.183424298	0.5399819	− 1.032674681
	PPARGC1A	− 0.220591156	− 0.021103317	**1.052226372**
	**PTGS2**^**#**^	− 0.445027435	**1.284844183**	0.692814698
	FGF12	− 1.679689073	0.070213602	− 0.73020918
Module 3	FGF16	− 1.720424769	− 0.320444214	− 0.7530489
	**FGF6**	− 0.667200674	− 1.845753277	**1.46974102**
	**FZD5**^**#**^	**2.578614852**	**1.546589322**	**1.0685415167**
	IL4	− 1.828811858	0.218179724	− 0.629046898
	**SDC4**	0.494492734	0.889929144	**1.041419575**
	SFRP5	0.302608269	0.179667321	− 1.770134417
	**SPP1**^**#**^	− 0.921871849	− 1.12673572	**2.802615419**
	WNT16	− 0.168834534	− 0.103897082	− 3.314461453

Up-regulated genes are highlighted in bold and downregulated genes in normal typeface.

*FC* fold change, *LD* longissimus dorsi muscle, *SB* semimembranosus, *PM* psoas major.

^#^The upregulated module genes identified as hub gene.

### Quantitative real-time PCR validation

**Figure 8:**
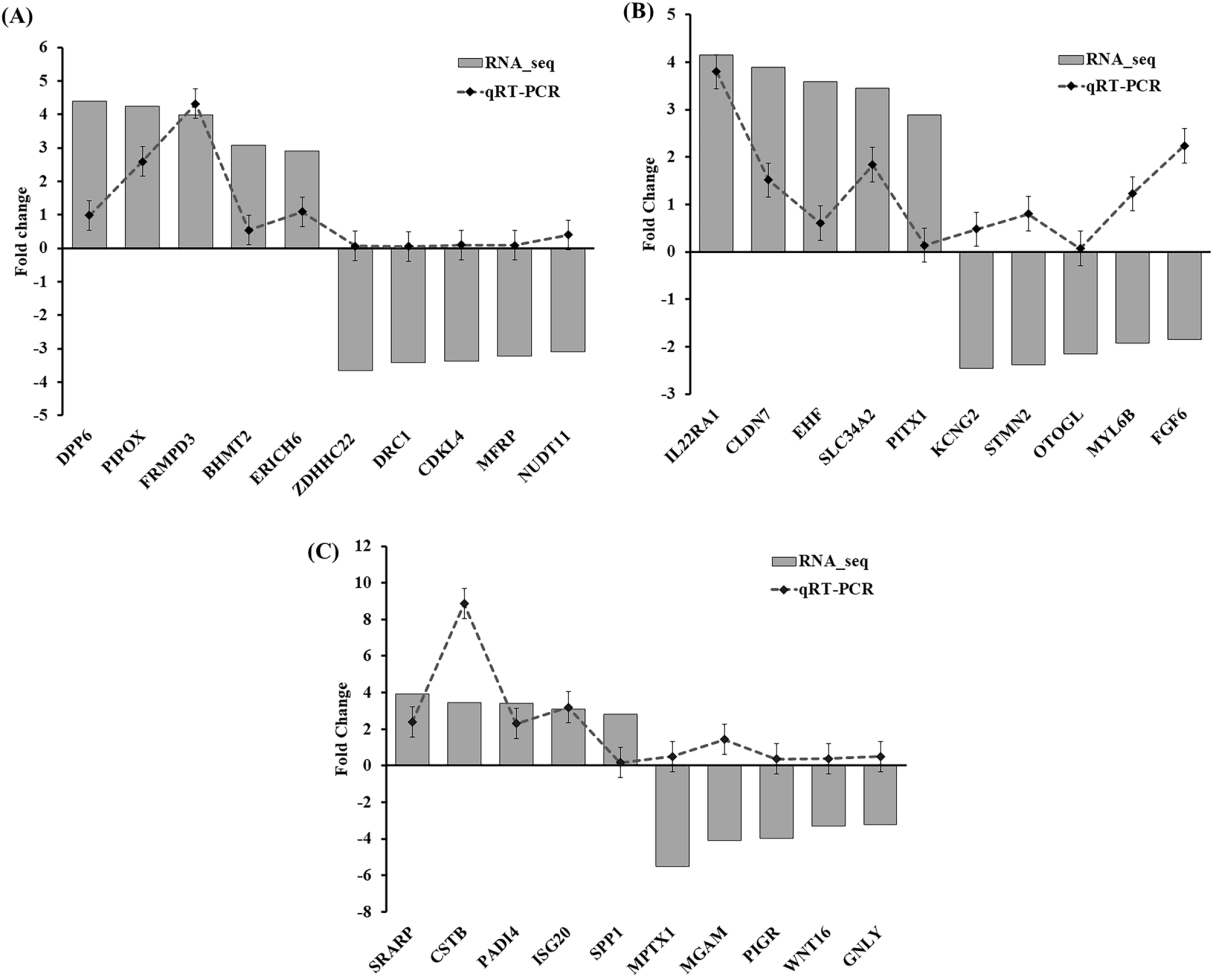
Verification of expression level of selected DEGs in the (**A**) LD, (**B**) SB, (**C**) PM muscle tissue samples by quantitative reverse‑transcription‑PCR (qRT‑PCR). Ten genes randomly selected from both up–regulated and down–regulated of each tissue were analyzed. The fold changes obtained from qRT‑PCR was compared with the fold changes of the RNA-seq result. *DEG* differentially expressed gene, *LD* longissimus dorsi, *SB* semimembranosus, *PM* psoas major.

To assess the reliability of the RNA-seq data, a subset of top five up-regulated and down-regulated DEGs was randomly selected for validation using qRT-PCR (Fig. 8). The qRT-PCR results demonstrated that the expression levels of up-regulated genes (*DPP6*, *PIPOX*, *FRMPD3*, *BHMT2*, *ERICH6*) and down-regulated genes (*ZDHHC22, DRC1*, *CDKL4*, MFRP, and *NUDT11*) in LD, up-regulated genes (*IL22RA*, *CLDN7*, *EHF*, and *SLC34A2*) and down-regulated genes (*KCNG2*, *STMN2*, *OTOGL*, *MYL6B*, and *FGF6*) in SB, up-regulated (*SRARP, CSTB*, PADI4, *ISG20*, and *SPP1*) and down-regulated (*MPTX1*, *MGAM*, *PIGR, WNT16,* and *GNLY*) in PM sample were significantly changed between high ADG and low ADG group in each tissue, which corroborated the findings from the RNA-seq analysis (Fig. 8).

## Discussion

Controlling factors that contribute to ADG is crucial for the Hanwoo beef industry, as a high occurrence of low weight can result in significant economic losses. Therefore, a comprehensive understanding of the genetic factors underlying key growth and economic traits of livestock is essential for identifying new genes and genetic pathways that can be leveraged through genomic selection to improve economic traits in livestock breeding programs.

Hence, the current study was carried out transcriptome analysis on these three LD, SB, and PM skeletal muscles to provide the novel molecular functions of the tissues, which may influence the ADG in Hanwoo steer. In South Korea, LD, the PM, and SB holds significant importance due to the high demand for steak. In Hanwoo beef, the LD and PM skeletal muscles, commonly referred to as top fillet and under fillet in the meat industry, stand out as highly coveted cuts of the carcass, possessing substantial commercial and culinary significance^11,12^. In our study, we selected male Hanwoo cattle with a significance difference in ADG group (high ADG ≥ 0.77 kg and low ADG ≤ 0.71 kg). The result of this study revealed that *DPP6* (LD), *IL22RA1* (SB), and *U6* (PM) genes were the top upregulated genes. Among these genes, *DPP6*, also known as dipeptidyl peptidase-like protein 6, is a gene that encodes a protein involved in neuronal signaling and ion channel regulation^13^. It has been suggested as candidate associated with altered lipid profile^14^, muscle atrophy in human^13^, and the rib eye Muscle Area in Hu Sheep^13^. According to Zhao et al., it is plausible that *DPP6* may have some influence on muscle physiology and potentially contribute to muscle wasting or atrophy^13^. *IL22RA1* (interleukin 22 receptor subunit alpha 1) has been shown to be receptor for interleukin-22 (*IL-22*), a cytokine involved in various biological processes, including cell proliferation and tissue regeneration in animals and human^15^. Therefore, *DPP6* gene can be suggested as one of good possible candidate gene for ADG variation in Hanwoo. While *IL22RA1*’s role in muscle growth is not extensively studied, there is evidence suggesting its secondary involvement in muscle physiology^15–17^. *IL22RA1* being identified as a candidate gene associated with health, adaptation and reproduction traits in cattle^18^. For PM tissue, small nuclear RNAs (U6) plays catalytic role at the core of the spliceosome^19^ but straight function associated to muscle development or growth performance in cattle not been reported before. Furthermore, a number of genes that were downregulated involved in diverse function linked to cell death, metabolism, cell growth and development were screened as possible candidate genes for ADG trait. Of the top downregulated DEGs, myosin light chain 6B (*MYL6B*), fibroblast growth factor 6 (*FGF6*), acyl-CoA synthetase bubblegum family member 1(*ACSBG1*), two pore channel 3(*TPC3*), polymeric immunoglobulin receptor(*PIGR*), Wnt family member 16 (*WNT16*), alpha-2-glycoprotein 1, zinc-binding (*AZGP1*), and forkhead box Q1(*FOXQ1*) genes were found to be involve in various function associated to growth, development and metabolism.

The total upregulated and downregulated significant DEGs were used to determine the GO and KEGG pathway analysis, which was then used to identify significant enriched pathways for the three tissues. Based on the pathway analysis, JAK-STAT signaling pathway was detected as the most significant enriched pathway in LD tissue. In muscle development and regeneration, this pathway plays a vital role in regulation of adipogenesis, process of fat cell development, controlling myoblast proliferation, differentiation, and the formation of muscle fibers^20–22^. A previous GWAS study identified candidate genes associated with the body weight trait in Chinese sheep, revealing their involvement in the JAK-STAT pathway^23^.

Among the upregulated DEGs in JAK-STAT pathway, we observed a significant increase in the expression of *CDKN1A* (cyclin dependent kinase inhibitor 1A) gene and common enrichment in conjunction with other pathways in our result. This gene is associated with the regulation of the G1/S checkpoint and is known to be crucial in cellular senescence during preimplantation embryo development in humans^24^. Zhang et al. reported that *CDKN1A* regulates myogenesis process in muscle stem cells^25^. Recently, *CDKN1A* gene has been reported as strong candidate gene for high fat milk production trait in dairy cattle (Chinese Holstein population)^26–28^. Moreover, another vital gene, *SOCS3* (suppressor of cytokine signaling family 3) known as negative regulator of JAK-STAT, was detected to be downregulated in our study^29^. It indicates that the downregulation of *SOCS3* could leads to *STAT3* phosphorylation which further may promotes lipid metabolism, cell cycle progression, differentiation^30,31^. Although future studies are expected to provide more comprehensive insights into the specific roles of both the genes in JAK-STAT pathway and other regulatory mechanisms in muscle growth, ultimately leading to weight gain. The findings indicate that the JAK-STAT pathway, along with other pathways, may affect the ADG trait in Hanwoo through its influence on myogenesis, lactation, cell growth and differentiation.

In SB tissue, the prolactin signaling pathway emerged as the most significant pathway. Here, our result revealed a significant upregulation of prolactin signaling pathway associated *PRLR* (prolactin receptor) gene by a log2FC of 3.05. The prolactin signaling pathway has significant involvement in the regulation of metabolism, immune system function, milk synthesis, cell growth, survival and pancreatic development. Activation of this signaling pathway was reported to promote cell growth and survival^32^. According to Chu et al., the *PRLR* gene closely related to prolactin signaling pathway is a candidate gene for prolificacy of small tail trait in han sheep^33^. In Egyptian Buffaloes, the *PRLR* has shown promise as a genetic marker for evaluating milk production and quality traits^34^. Additionally, another gene associated with the prolactin signaling pathway, *MAPK13* (mitogen-activated protein (MAP) kinase family), exhibited downregulation. *MAPK13* encodes a protein belonging to the mitogen-activated protein (MAP) kinase family, which serves as a convergence point for various biochemical signals^35^. MAP kinases are involved in numerous cellular processes including proliferation, differentiation, transcription regulation, and development^35,36^. Therefore, it can possibly suggest that *PRLR* and *MAPK13* gene can be important possible biomarker associated to high ADG in Hanwoo.

Subsequently, the KEGG analysis identified PPAR signaling pathway as a potential candidate pathway that was the top most significant pathway found from the transcriptome analysis of PM tissue. Further, we identified the major downstream pathways associated with PPARs signaling pathway such as adipocytokine signalling pathway and regulation of lipolysis in adipocytes. The PPAR signaling pathway has been examined in many studies with regard to its function in adipose tissue influences adipogenesis, glucose metabolism, and adipokine secretion in cattle^8,37^. Recently, He et al., described that 13 tag single nucleotide polymorphisms in the PPAR signaling pathway are associated with porcine meat quality traits^38^. A study on Hanwoo cattle investigated the gene expression patterns associated to PPAR signaling pathway. The results of this study demonstrated a significant association between PPAR signaling and genes involved in fatty acid oxidation. This association ultimately led to an increase in triglyceride formation through ATP production^8^. A muscle transcriptome study conducted earlier identified the adipocytokine signaling pathway and PPAR signaling pathway as potential candidate pathways associated with the longissimus thoracis muscles in meat-type sheep from India^39^. Hence, it can be hypothesized that the PPAR signaling pathway plays a role in controlling adipogenesis and influencing the capacity of adipose tissue to store lipids, thereby affecting the fat mass in Hanwoo muscles. Consequently, by regulating the size and quantity of adipocytes within the muscle, this pathway may impact the average daily gain characteristic in Hanwoo cattle.

In our study, the genes involved in PPAR signaling pathway were *ADIPOQ* (Adiponectin, *C1Q* and collagen domain containing), *PCK2* (Phosphoenolpyruvate carboxykinase 1), *FABP4* (Fatty acid binding protein 4, adipocyte), *PLIN1* (perilipin). *FABP4* gene reported to be facilitates the uptake of fatty acids and their intracellular trafficking, contributing to the process of adipocyte maturation and lipid accumulation^8,40^. *ADIPOQ* is a well-known homeostatic factor for regulating glucose levels, and lipid metabolism and reported to be candidate gene for carcass trait^41^. *PLIN1* plays a crucial role in controlling the levels of triglycerides and the size of lipid droplets in adipocytes, contributing to the maintenance of lipid balance^42^. It is also suggested as possible robust candidate gene marker for body weight in cattle breeding programs^43^. Furthermore, the *SLC2A1* gene enriched in adipocytokine signaling pathway was upregulated by more than twofold here in PM tissue. *SLC2A1*, a glucose transporter, has also been implicated in facilitating the transport of fatty acids^44^. In summary, it is postulated that the mentioned genes may facilitate the accumulation of intramuscular fat in cattle by controlling the synthesis, transport of fatty acids, lipid balance by regulating the respective signaling pathways and potentially affecting the ADG trait. However, a more comprehensive understanding of the precise regulatory mechanisms involved requires further exploration in future studies.

Our GO analysis revealed that DEGs of LD tissues were mainly enriched in branching involved in labyrinthine layer morphogenesis, retinoic acid metabolic process, T cell chemotaxis and detoxification of copper ion biological process. For the SB tissue, we found significant biological process GO terms that are the sodium ion transmembrane transport and response to estrogen biological process. In PM tissue GO analysis, the result revealed some important biological process GO terms that are interleukin production, fat cell differentiation, carbohydrate metabolic process, ion transmembrane transport, insulin resistance and etc. Among all these GO terms, ion transmembrane transport facilitates nutrient absorption, carbohydrate metabolism provides energy, and fat cell differentiation contributes to energy storage, all of which are essential processes for weight gain in cattle. These biological processes have been reported to be have dominance effect on porcine and cattle final weight and back fat thickness^18,45,46^. Moreover, the GO network analysis revealed that several essential genes, which were commonly shared among significant GO terms, exhibited significant upregulation. For example, *FZD5* (frizzled class receptor 5) involved in two different GO terms are found to be upregulated in our datasets. These genes likely to play crucial roles in the biological processes, cellular components, or molecular functions represented by the enriched GO terms. Further investigation and validation of these genes can provide insights into their specific roles and mechanisms for ADG in Hanwoo cattle.

Furthermore, we constructed a PPI network to gather valuable interaction information regarding the differentially expressed genes (DEGs). The PPI network in the current study was created with total significant DEGs (P-value of ≤ 0.05, log2FC of ≥ 1.5) collected from three experimental tissues. In present investigation, we identified 12 hub genes that are *JUN* (transcription factor Jun), *CDKN1A*, *CDH1* (cadherin-1), *TP53* (tumor Protein P53), *FZD5*, *PPARG* (Peroxisome proliferator-activated receptor gamma), *LEP* (leptin), *IL4* (interleukin 4), *PTGS2* (prostaglandin-endoperoxide synthase 2), *AXIN2* (axin-related protein 2), *CDH2* (cadherin-2) and *MYC* (MYC proto-oncogene). These findings highlight the centrality of these hub genes and suggest their involvement in critical biological processes or pathways associated with growth performance in Hanwoo. By integrating the DEGs from these tissues, the PPI network was generated to capture correlation patterns of gene expression across samples, the new interactions and relationships between proteins that tend to be co-regulated or functionally related^47^.

Among all the hub genes, notably the *CDKN1A* and *FZD5* were spotted to be upregulated (> onefold) in all the three experimental tissue samples. In our study, we have identified *CDKN1A* as a key hub gene that works as inhibitor in cell cycle and control myogenesis process in muscle stem cells^25^. Furthermore, our module analysis indicated that *CDKN1A* is associated with module 1. Interestingly, in our current experiment, we observed that *CDKN1A* is enriched in various pathways, including those related to different types of cancers, Hepatitis B, Epstein-Barr virus infection, PI3K-Akt signaling pathway, p53 signaling pathway, and JAK-STAT signaling. *CDKN1A* has been described as candidate gene associated with carcass quality trait and milk production in beef cattle^48^. These findings along with our pathway analysis result collectively suggest that *CDKN1A* may influence muscle growth in Hanwoo cattle by regulating the muscle differentiation at the myogenin step, thereby subsequently affecting the ADG trait. However, more research is needed to confirm this theory. The other gene, *FZD5* is a protein coding gene, which functions as transmembrane receptor and mediates Wnt ligands binding in Wnt signaling pathway and plays role in the control of tissue regeneration, cell proliferation and cell differentiation^49,50^. One recent study has proposed that the activation of *FZD5* by Wnt protein could potentially play a role in regulating adipose differentiation^49^. A previous GWAS study identified *FZD5* as a candidate gene associated with fertility traits in Beef Heifers^51^. They suggested that the *FZD5* gene plays a role in the WNT pathway, which aligns with our findings and provides additional support for our data^51^. Therefore, it could be valuable to study further on *FZD5* functions in the muscle growth and ADG trait.

In current study, the top three modules were selected for further investigations of PPI network. Our result presented that the genes in all the modules are involved in several important signaling pathways including PI3K-Akt signaling pathway, Wnt signaling pathway, JAK-STAT signaling pathway, PPAR signaling pathway, IL-17 signaling pathway, and C-type lectin receptor signaling pathway. PI3K-Akt signaling and Wnt signaling pathway were found to be common in two modules i.e. module 1 and module 3. A considerable number of studies have reported that the PI3K-Akt signaling and Wnt signaling pathways are the key pathways for regulating cell propagation, adipose proliferation, fat deposition, and muscle development in bovine thus subsequently effect the meat production and carcass traits in cattle^52–55^. Notably, the outcomes from GO/KEGG analysis were matched to the functional annotations of genes in the most top three modules of PPI network. Additionally, it is noteworthy to mention that genes enriched in module 1 (*CDKN1A*, *AXIN2*, *CDH2*, *MYC*, and *TP53*), module 2 (*JUN, PPARG, PTGS2)*, and module 3 (*TP53, FZD5, IL4,* and *MYC*) were found as hub genes as well. The speculation that the identified genes, which are differentially regulated and associated with various pathways in different tissues, could be potential candidate genes affecting the ADG trait is reasonable. However, it is crucial to note that further intensive investigations are necessary to validate and confirm the significance of the identified candidate genes and pathways.

Some limitations are associated with this study. Firstly, the sample size was relatively small and the age difference between low and high group reaches up to 168 days. Additionally, our research was exclusively conducted on only three skeletal muscles. One advantage of this approach is the ability to obtain results from cattle with consistent or uniform characteristics. This study is also the first to report the use of three different skeletal muscles for ADG trait in Hanwoo cattle. A larger scale transcriptome study is needed on different traditional adipose depots subcutaneous (SQF), visceral (VAT), intermuscular (INTMF), intramuscular (IM), and bone adipose for better understanding of molecular mechanism of ADG in cattle^56^.

## Conclusion

To sum up, our study involved a comparative analysis of three different skeletal muscle transcriptomes in Hanwoo cattle that exhibited varying levels of average daily weight gain. A total of 200, 172, and 210 DEGs were screened from LD, SB and PM muscle tissues, respectively. Through functional enrichment, hub gene, and module analysis of DEGs in three distinct muscle tissues, we have identified several possible candidate genes, namely *DPP6*, *CDKN1A*, and *FZD5*, along with significant pathways, including the JAK-STAT signaling pathway, prolactin signaling pathway, and PPAR signaling pathway. These findings suggest a crucial involvement of these candidate genes and pathways in influencing the variation of average daily gain (ADG) in Hanwoo cattle. To fully comprehend their specific mechanisms related to carcass weight traits, further investigations are warranted.

## Materials and methods

### Animals, phenotypes, and sample collection

The National Institute of Animal Science in the Republic of Korea's Animal Care and Use Committee gave its approval to all experimental techniques mentioned in this paper (Approval No. NIAS-2022133). The National Institute of Animal Science's (NIAS) Animal Genomics and Bioinformatics Division assembled the tissue samples of Hanwoo cattle for the dataset used in this study. Also, all the experiments in the manuscript follows the recommendations in the Animal Research Reporting in Vivo Experiments (ARRIVE) guidelines. At the slaughterhouse of the National Institute of Animal Science, the cow was stunned with a captive bolt gun and then slaughtered as per American Veterinary Medical Association​ (AVMA) Guidelines for the Euthanasia of Animals. The average daily weight gain (ADG, kg/d) was examined and calculated the ratio as following-

1 $${\text{ADG}}\left({\text{kg}}/{\text{d}}\right)=\frac{\mathrm{Body\,weight }\left({\text{kg}}\right)\mathrm\,{at\,Slaughter\,day}-\mathrm{Initial\,Born\,weight}\,({\text{kg}})}{{\text{Age}}\left({\text{days}}\right)}$$

In this study, 14 cattle used were at growing stage which corresponds to the early fattening stage. Additionally, these cattle were assigned to the same feed and all the cattle were weaned at the same year. Post-slaughter muscle samples including the longissimus dorsi muscle (LD), rump semimembranosus (SB), and psoas major (PM) were collected. Sterile surgical techniques were used, such as washing the muscle collection site with 70% ethanol and using a sterile scalpel to make skin incision^7^. The sample integrity was preserved through immediate preservation in liquid nitrogen and subsequent storage at − 80 °C.

### RNA isolation and sequencing

The total RNA was isolated from each homogenized tissue sample (LD, SB, and PM) using the Qiagen RNeasy kit (Qiagen, Hilden, Germany) following the manufacturer's instructions. The RNA's purity and integrity were assessed using a nano photometer (IMPLEN, CA, USA) and the RNA Nano 6000 Assay kit on the Bioanalyzer 2100 equipment (Agilent Technologies, CA, USA). Samples with RNA integrity number (RIN) values exceeding 8 were selected for library preparation. The NEBNext UltraTM RNA Library Prep Kit for Illumina (NEB, Iswich, MA, USA) was employed to prepare the library, involving random fragmentation, adapter ligation, and tagmentation as per the manufacturer's guidelines. The sequencing was performed using the Illumina NovaSeq 6000 platform. The adapter-ligated fragments were amplified via PCR, followed by gel purification. Cluster generation was achieved using the TruSeq PE cluster kit v4-cBot-HS (Illumina) following the manufacturer's instructions. For sequencing, the library was loaded into the Illumina NovaSeq 6000 System, which produced paired-end reads. Base calling on the raw images generated by the Illumina sequencer was performed using real-time analysis (RTA) software. Finally, the generated BCL image files were converted to FASTQ raw readings using the Illumina BCL to fastq program, which were then used for post-analysis.

### RNA sequencing data processing and analysis

For the data analysis in this study, the input was derived from FASTQ files. The quality of the raw RNA-seq data was assessed using FastQC v0.11. Trimmomatic v0.39 was utilized to perform trimming of N bases, removal of adapters from the 5' and 3' ends, and filtering of reads with a quality score below Q20 for quality control during the generation of raw reads with the paired-end option. The filtered sequences were mapped to the *Bos taurus* reference genome (version ARS-UCD 1.2) using the Hisat2 v2.2.1 mapping program with the corresponding index file. Subsequently, the mapped reads were used to generate a count matrix using FeatureCounts from the Subread package (v2.0.1). A PCA plot without any batch correction was then generated to visualize the data. Finally, the differential expression analysis of genes (DEGs) was performed in R (v3.4.4) using each read counts matrix file.

### Differential gene expression (DEG) analysis

DESeq2 package in R was utilized to perform differential expression (DE) analysis between the low ADG and high DAG groups of LD, SB, and PM muscle tissues. DEGs were identified using a P-value threshold of less than 0.05 and a log2fold change equal to or higher than 1.5^47,57^. The resulting DEGs were classified into up-regulated and down-regulated genes based on their log2fold change values. To visualize the genetic distinctions between the two groups, a heatmap was constructed using the pheatmap package (v1.0.12), accessible at https://cran.r-project.org/web/packages/pheatmap/pheatmap.pdf. Furthermore, the PCA plot and volcano plot were generated using ggplot2 (v3.3.3) and the VolcaNoseR web tool (https://github.com/JoachimGoedhart/VolcaNoseR/issues)^58^, respectively.

### Functional and pathway enrichment analysis of DEGs

GO and KEGG pathway enrichment analysis of the DEGs linked to ADG deviation was performed using the DAVID web tool^59,60^. The KEGG graphs were obtained from www.kegg.jp/kegg/kegg1.html^61^. The background parameter for the investigation was the genome of the cow (*Bos taurus*). For the purpose of determining statistically significant differences, a significance cutoff of P-value ≤ 0.05 was chosen. Cytoscape (v3.2.1) and the ClueGO plugin were used to build and display the enriched pathways network^62^.

**Table 6:** List of primer used in this study for qRT-PCR verification of RNA-seq results.

Gene symbol	F-primer (5ʹ—3 ʹ)	R-primer (5ʹ—3 ʹ)	
LD tissue			
DPP6	TACCTGAGCACCTACCTCCT	TCGGTTGTCAAGTCCATGGA	Up-genes
PIPOX	CTGGCAAGAGAAGGTTCCTG	CATTGTTGCCGTGGTGATAG	
FRMPD3	GAGGAGGACGTGAGTGAAGC	ATCGGACCTTCACAGGATTG	
BHMT2	TCTGGATAGTGGGGAGGTTG	TACAGCTTCCCACAGGCTTT	
ERICH6	GCGGAGAAAACAAGAACGAC	TCCCGTCTGGAAATGAAGTC	
ZDHHC22	CTGGGCAACTATGTCCTGGT	GCGAGGTGTAGAGGCAGAAC	Down-genes
DRC1	GGATGGGAAGTCACAGGAGA	TGGCAGGGATAACCGTAGTC	
CDKL4	GGCAAACACTCCAAGCTCTC	TATCCCCCACAAGAAGTTCG	
MFRP	GTGCCAAGTTTTCAGGGTGT	GGCTGAAGTTGTGGAAGAGC	
PLPPR3			
SB tissue			
IL22RA1	CCACAGACAAGGGTCCAAGT	CCTCGGAACAGAGAGTCCAG	Up-genes
CLDN7	TGCCTTGATAGCTTGCTCCT	CGGTATCCAGCTTTGCTCTC	
EHF	ATGAGTCTGCAGGAGTTCAC	GCTACCATAGTTGGTGTCGT	
SLC34A2	AGCCTGAGAAAGCTAAGGAG	CTCTCTCTGACCACTTGAGC	
PITX1	CAGCTCCATCTCCTCCAT	AGTGCTGCTTAGACTTGAGC	
KCNG2	GAGTGCTCCCCTAAGTGTCG	ACACGTAGAAGGGCAGGATG	Down-genes
STMN2	ATCTGCTCTTGCTTCTACCC	GCCTCCTGAGACTTTCTTCT	
OTOGL	CACAGGCTTCCACACTTTGA	CATTGCCCCTTCATCAGTCT	
MYL6B	TCCAGGGTTGAGCTACCATC	TATCCCAAGACCCTGAGCAC	
FGF6	CCCAGCTTCCAAGAAGAGTG	CTAGGGAGGAAGTGCGTGAC	
PM tissue			
SRARP	GAACCTGGAGACCAGCTCAG	GAGCCTCCAGAGTCAGGTTG	Up-genes
CSTB	CAGCTGGAGGAGAAGGAGAA	GGTCTGGTAGCTGGTCAAGG	
PADI4	CCAGGAGTGGTTGTGAAGGT	GCACTCAGGGAGATTTCGAC	
ISG20	ACTATAGGACCCCGGTCAGC	GGCGTAGTCGCTCATGTTCT	
SPP1	GATGGCCGAGGTGATAGTGT	GTTGGTTTCCTTGCTGTGGT	
MPTX1	CCTGAAAGCCTTCACAGACC	TCCCAGCTGACACAGAGATG	Down-genes
MGAM	TGCCTTGACATACCGTACCA	CCATAGCGACACAGCTGAAA	
PIGR	ACCATCAACTGCCCTTTCAC	TGAGCTTGACTCGGTTGATG	
WNT16	CATGACCGAGTGTTCCTGTG	CTGCCTTCCAGCTTCATTGT	
GNLY	AGGAGAAGAGCTGGGCCTAC	TTTCCAGCTGTGATGTCTGC	

*LD* longissimus dorsi muscle, *SB* semimembranosus muscle, *PM* psoas major muscle.

### Hub genes, co-expression module and pathway detection by PPI network and module analysis of DEG

The online database Search Tool for the Retrieval of Interacting Genes/Proteins (STRING, Version 10.0) was used to build the protein–protein interaction (PPI) network of the differentially expressed genes (DEGs). We have collected all the significant DEGs from each tissue and submitted to STRING database for constructing the network. To identify meaningful interactions, an interaction score of 0.4 was used as cut-off value. The Cytoscape program was used to visualize the PPI network^47^. For the identification of key genes in the PPI analysis, we utilized the CytoHubba plugin within Cytoscape^63^. We applied four different ranking approaches provided by CytoHubba: maximal clique centrality (MCC), edge percolated component (EPC), maximum neighborhood component (MNC), and node connect degree. From each ranking approach, we extracted the top 20 genes. The hub genes were then determined as the intersecting genes among the results of all four CytoHubba ranking approaches. By using this intersection approach, we were able to identify the hub genes that were consistently highly ranked across multiple methods, indicating their potential importance in the PPI network.

To identify densely connected co-expression modules within the protein–protein interaction (PPI) network, we employed the Molecular Complex Detection (MCODE) plugin in Cytoscape^64^. The MCODE parameters used included a degree cut-off of 2, a node score cut-off of 0.2, a K-score of 2, and a maximum depth of 100.

### Quantitative reverse‑transcription‑PCR (qRT‑PCR)

We carried out qRT-PCR on top five arbitrarily selected upregulated and downregulated chosen differentially expressed genes (DEGs) in order to confirm the results from RNA-seq. Using the Primer3 tool (Version 0.4.0), primer design for qRT-PCR was completed^65^. Using the housekeeping gene GAPDH (Glyceraldehyde 3-phosphate dehydrogenase), the mRNA levels of the DEGs were adjusted. Using the 2^−ΔΔct^ technique, relative gene expression values were calculated. In addition, the expression levels of regulated genes were compared between the RNA-seq and qRT-PCR data. Table 6 contains the primer sequences for the relevant genes and the reference gene.

### Statistical analysis

qRT‑PCR experimental results are expressed as mean ± standard error of measurement (SEM). GraphPad Prism 6 software (GraphPad Software, San Diego, CA, USA) was used to analyze the gene expression data and ADG data between two groups with a pairwise t-test. *** signifies P < 0.001 and * displays P < 0.05 as statistical significance.

### Ethics declarations and consent to participate

This study was approved by the Institutional Animal Care and Use Committee (IACUC) of the National Institute of Animal Science (approval no. NIAS2022133). All the experiments were performed in accordance with relevant guidelines and regulations of Institutional Animal Care and Use Committee (IACUC) of the National Institute of Animal Science.

### Approval for animal experiments

All the experiments in the manuscript follows the recommendations in the ARRIVE guidelines.

### Supplementary Information

Supplementary Legends.

Supplementary Figure S1.

Supplementary Figure S2.

Supplementary Figure S3.

Supplementary Figure S4.

Supplementary Table S1.

Supplementary Table S2.

## Data Availability

The sequencing raw reads were deposited in NCBI GEO portal to the sequence read archive (SRA) with accession number PRJNA1002884.
