# Supplementary material for: A transcriptomic analysis of skeletal muscle tissues reveals promising candidate genes and pathways accountable for different daily weight gain in Hanwoo cattle
Source: Sci Rep. 2024 Jan 3;14:315. doi: 10.1038/s41598-023-51037-9 (PMC10764957; doi:10.1038/s41598-023-51037-9)
Supplement: Supplementary file 1 — Supplementary Legends. [file 41598_2023_51037_MOESM1_ESM.docx]

**Supplementary Figure Legends**

**Supplementary Figure 1.** Illustrating of significant difference in phenotype between high and low ADG group.

**Supplementary Figure 2**. Illustrating of correlation between the expression levels of tissues. LLD, low ADG-longissimus dorsi; HLD, high ADG-longissimus dorsi, LSB, low ADG-semimembranosus; HSB, high ADG-semimembranosus; LPM, low ADG-psoas major; HPM- high ADG-psoas major. ADG, average daily weight gain.

**Supplementary Figure 3**. Showing the top enriched KEGG Pathway map in (A) LD, (B) SB, (C) PM muscle tissue samples. The KEGG graph was obtained from www.kegg.jp/kegg/kegg1.html. LD, longissimus dorsi; SB, semimembranosus; PM, psoas major; KEGG, Kyoto Encyclopedia of Genes and Genomes.

**Supplementary Figure 4**. Protein-protein interaction (PPI) network for DEGs with P ≤ 0.05 and log2FC ≥ 1.5 of three tissues. A total of 363 nodes and 902 interaction associations were detected. DEGs, differentially expressed genes; FC, fold change.
