# Supplementary figures and images for: A transcriptomic analysis of skeletal muscle tissues reveals promising candidate genes and pathways accountable for different daily weight gain in Hanwoo cattle
Source: Sci Rep. 2024 Jan 3;14:315. doi: 10.1038/s41598-023-51037-9 (PMC10764957; doi:10.1038/s41598-023-51037-9)

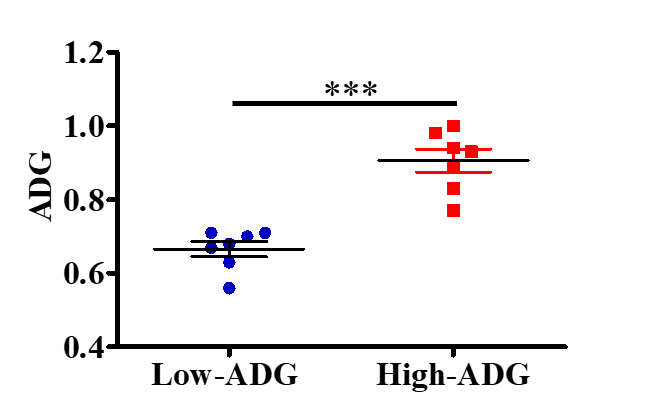

Supplement: Supplementary file 2 — Supplementary Figure S1. [file 41598_2023_51037_MOESM2_ESM.tif]

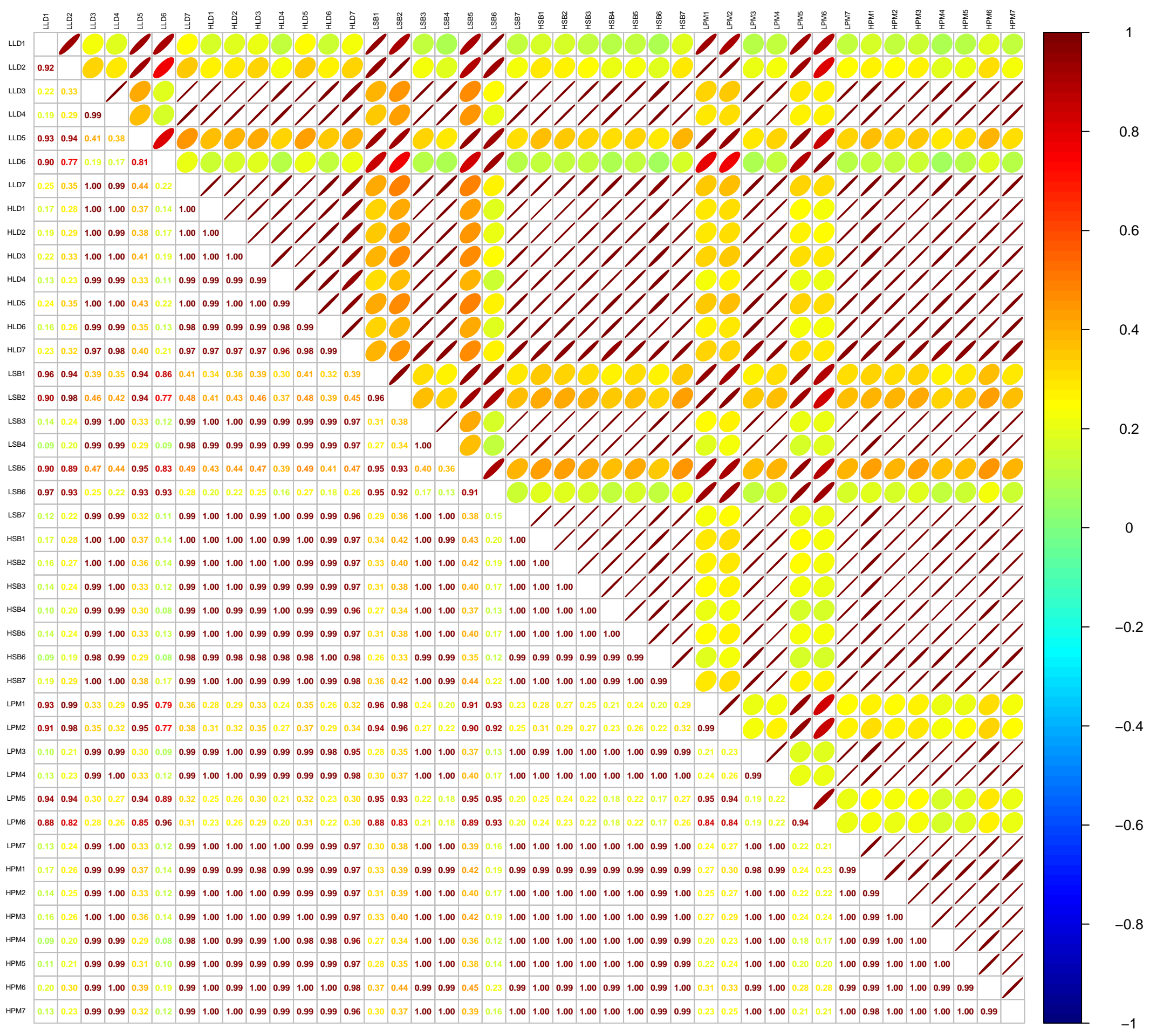

Supplement: Supplementary file 3 — Supplementary Figure S2. [file 41598_2023_51037_MOESM3_ESM.pdf]

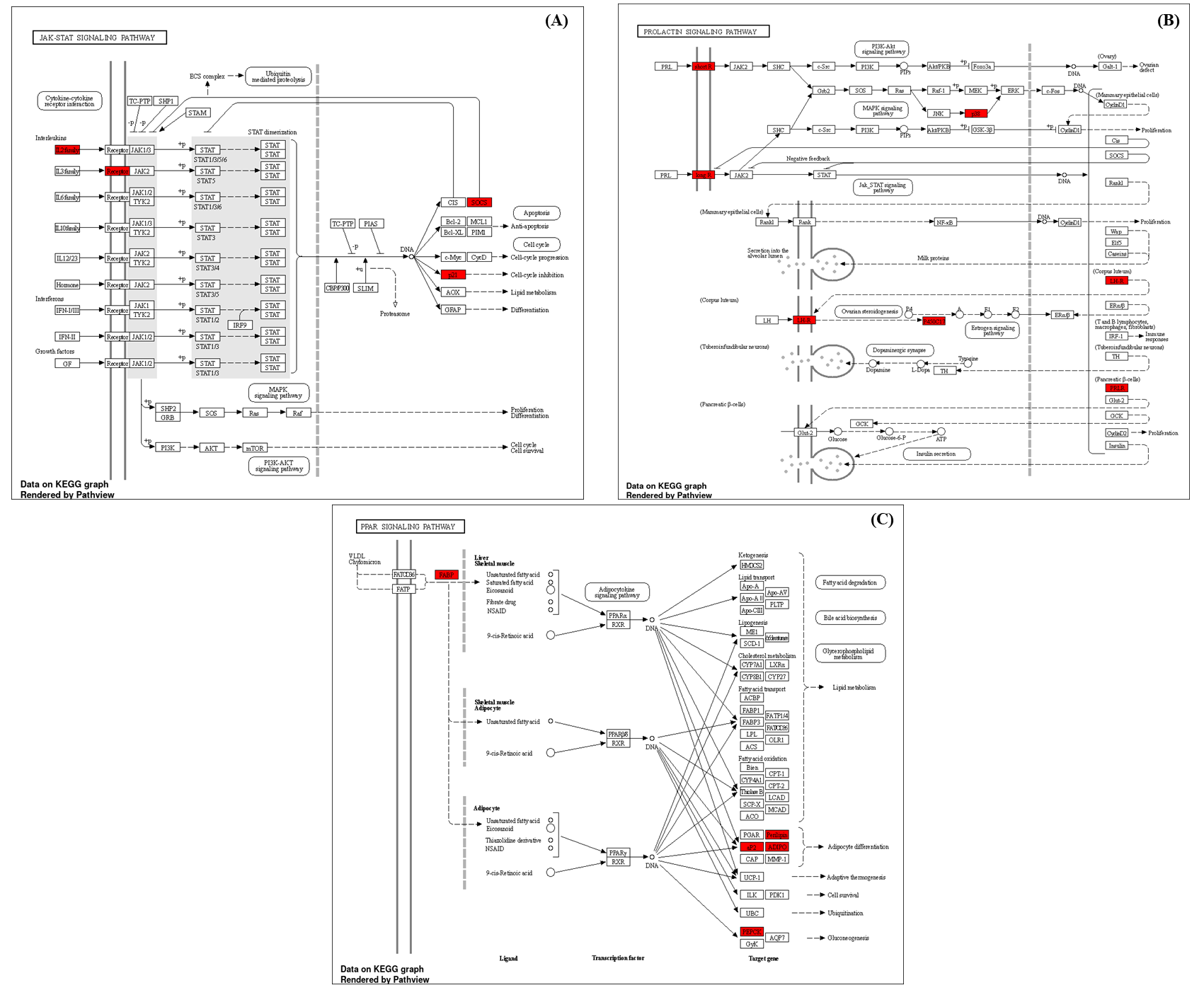

Supplement: Supplementary file 4 — Supplementary Figure S3. [file 41598_2023_51037_MOESM4_ESM.tif]
